# Q Fever—A Neglected Zoonosis

**DOI:** 10.3390/microorganisms10081530

**Published:** 2022-07-28

**Authors:** Qudrat Ullah, Tariq Jamil, Muhammad Saqib, Mudassar Iqbal, Heinrich Neubauer

**Affiliations:** 1 Faculty of Veterinary and Animal Sciences, The University of Agriculture, Dera Ismail Khan 29111, Pakistan; 2 Institute of Bacterial Infections and Zoonoses, Friedrich-Loeffler-Institut, 07743 Jena, Germany; heinrich.neubauer@fli.de; 3 Department of Clinical Medicine and Surgery, Faculty of Veterinary Science, University of Agriculture Faisalabad, Faisalabad 38000, Pakistan; 4 Department of Pathology, Faculty of Veterinary and Animal Sciences, The Islamia University of Bahawalpur, Bahawalpur 63100, Pakistan; mudassar.iqbal@iub.edu.pk

**Keywords:** *Coxiella burnetti*, coxiellosis, Q fever, Pakistan

## Abstract

Q fever remains a neglected zoonosis in many developing countries including Pakistan. The causing agent *Coxiella (C.) burnetii* is resistant to environmental factors (such as drying, heat and many disinfectants), resulting in a long-lasting infection risk for both human and animals. As the infection is usually asymptomatic, it mostly remains undiagnosed in animals until and unless adverse pregnancy outcomes occur in a herd. In humans, the infection leads to severe endocarditis and vascular infection in chronic cases. Limited data are available on molecular epidemiology and evolution of this pathogen, especially in ruminants. Genomic studies will help speculating outbreak relationships in this scenario. Likewise, pathogenesis of *C. burnetii* needs to be explored by molecular studies. Awareness programs and ensuring pasteurization of the dairy milk before human consumption would help preventing Q fever zoonosis.

## 1. History and Background

“Q” fever was first reported in 1935 as an outbreak of febrile illness of unknown origin with flu-like symptoms in abattoir workers by Dr. E. H. Derrick, Director of the Laboratory of Microbiology and Pathology, Queensland Health Department in Brisbane, Australia [1]. A *Rickettsia*-like organism was isolated from experimentally infected mice spleen [2]. Meanwhile in Hamilton, Montana, a suspected “Nine Mile agent” was isolated from *Dermacentor andersoni* ticks collected from Nine Mile, Montana, which showed properties of both virus and rickettsia [3]. A possible relation was described between these two organisms when a patient in Montana developed an illness while handling “Q fever” infected samples sent from Brisbane [4]. Initially it was named *Rickettsia burnetii,* but was then renamed to *Coxiella (C.) burnetii* [5]. An influenza-like infection “Balkangrippe” was also reported in soldiers from the Balkan regions in 1940 and in German and American troops during World War II (1939–1945). It was later identified as *C. burnetii* infection [6,7]. Other synonyms for Q fever may include Australian Q fever, abattoir fever, Balkan influenza, nine-mile fever and pneumorickettsiosis [7,8]. Currently, the term ”Q fever” is more associated to infection in humans, whereas “Coxiellosis“ is applied to animals [9,10].

Q fever is a widespread zoonosis present almost worldwide [11,12]. It is strictly an intracellular bacterium and has a wide range of hosts including ticks, fish, reptiles, birds, ruminants and humans [13]. Sheep and goats are considered as reservoir hosts and a risk for human infection. Hence, for proper control and prevention of Q fever infection in humans, animals and the environment, it is important to understand the disease in ruminants [14,15]. Aerogenic transmission is the major route for dissemination via contaminated air or dust [16,17]. A recent outbreak of Q fever in the Netherlands was linked to wind dispersion from a site where infected goats were kept [18]. In ruminants, the organism enters the blood stream and reaches its predilection sites, i.e., placental membranes, supramammary lymph nodes and mammary glands, where it resides and multiplies [10,19]. The ability of *C. burnetii* to multiply within the lysosomal vacuole inside phagocytic cells and variation in the lipopolysaccharide (LPS) antigen during Phase I and Phase II are some unique features that make it distinctive from other bacteria [20]. *C. burnetii* can exit in two different morphological forms. The first one is metabolically dormant SCV (small cell variant), which is a highly resistant form of *Coxiella*, and the other form is LCV (large cell variant), which is the metabolic active form of *C. burnetii* present within the host cell [21,22,23].

According to the World Health Organization (WHO), brucellosis, Q fever and Rift Valley fever are neglected zoonotic diseases (NZDs) which can be prone to misdiagnosis and under-reporting [24,25]. The US Center for Disease Control and Prevention (CDC) classified this bacterium as a category B biological agent due to its acute debilitating-disease-causing nature in targeted human populations [21,26,27].

### 1.1. Coxiella burnetii—The Etiological Agent

*C. burnetii* is a Gram-negative, strictly intracellular, pleomorphic bacterium ranging in size from 0.2 to 0.5 μm in width and 0.4–1.0 μm in length. It belongs to domain Bacteria, phylum Proteobacteria, class Gammaproteobacteria, order Legionellales, family Coxiellaceae, genus *Coxiella* and species *C. burnetii* [22,28,29]. The incubation period in humans is quite variable (2–4 weeks or even more) depending upon the inoculation dose, route for infection and antigenic phase of *C. burnetii*. An important characteristic of this pathogen is the presence of a structurally and antigenically distinctive lipopolysaccharide (LPS) molecule in its cell wall [22]. Based on the structure of LPS, it exists in two distinct antigenic forms: Phase-I and Phase-II. Phase-I is a virulent form having full LPS and is isolated from infected hosts. Phase II is a virulent form of *C. burnetii* having an incomplete or truncated LPS molecule without a terminal O-antigen. Phase II is obtained by repeated passages of Phase I in embryonated eggs or in cell cultures. In the *C. burnetii* genome, a 38 kb region encodes for LPS, which is responsible for its antigenic phase variation by chromosomal deletions [30,31,32]. Similarly, *C. burnetii* can exist in two different morphological forms that can be differentiated under an electron microscope, i.e., a large cell variant (LCV) and a small cell variant (SCV). LCV is the large, bacillus form and metabolically active, while SCV is small, coccoid and metabolically inactive. SCV is resistant to environmental stress and can survive longer in harsh environments [8,28,33].

The *Coxiella* genus includes other *Coxiella*-like organism species, e.g., *C. cheraxi* found in crayfish and a novel *Coxiella*-like organism present in birds and ticks. *C. cheraxi* has the highest genetic homology with *C. burnetii*. *Candidatus* Coxiella avium is another novel pleomorphic *Coxiella*-like organism isolated from birds. It multiplies within the acidic vacuole of host macrophage cells, leading to systemic infection and mortality. Similarly, *Coxiella*-like endosymbionts (CLE) are also present in ticks [20,27]. The virulence of *C. burnetii* is associated with a type of the strain involved in the disease. Four different lines of *C. burnetii* were found by the multispacer sequence typing (MST) technique. Sequence type (ST) 8 was isolated from sheep in France, ST 15 from goats in France, ST 16 from ticks in Montana (USA) and ST 20 from cattle in France [34].

### 1.2. Isolation and Propagation

Isolation and propagation is not the commonly used technique for routine diagnosis of *C. burnetii* due to its biohazard potential. It is time consuming and biosafety level (BSL) 3 laboratories are required. Moreover, technical expertise is also required. Isolation remains important for phenotypic and genotypic characterization of *C. burnetii* by multilocus variable number of tandem repeats analysis (MLVA) and multispacer sequence typing (MST), etc. Sample quality, condition and concentration of the pathogen in a sample are the factors that strongly affect the success of isolation and propagation [20,35,36]. Although *C. burnetii* is resistant to environmental stress and can survive outside the host for a long period, it requires host cells for intracellular multiplication [37]. Different methods are used for isolation and propagation of *C. burnetii*, including inoculation of embryonated chicken eggs, cell culture, laboratory animals or use of a novel axenic medium [38,39].

#### 1.2.1. Embryonated Egg Inoculation 

Embryonated egg inoculation has been traditionally used for direct isolation and propagation of *C. burnetii*. In this technique, the egg yolk of a 6–7 day old embryonated chicken egg is inoculated in a sterile environment. Usually, after 10–15 days of incubation, the yolk sac is harvested. Stained smears of the yolk sac wall are observed microscopically to assess the presence of *C. burnetii* and absence of bacterial contamination. A typical straw yellow color with white spots develops in infected yolk sacs, while uninfected yolk sacs appear orange with viscous consistency. Polymerase chain reaction (PCR) is used to confirm the *C. burnetii*-DNA [20,31].

#### 1.2.2. Cell Culture

Since *C. burnetii* is an intracellular pathogen, standard biological media are not suitable for its growth [40]. A cell culture system, known as shell vial cell culture for isolation and propagation of obligate or facultative intracellular bacteria such as *C. burnetii*, has been described [41]. In this system, a suspension containing *C. burnetii* infected material is inoculated into human embryonic lung (HEL) fibroblast cells grown within eggshell vials on 1 cm^2^ cover slips. The HEL fibroblasts are the cells used most for isolation and propagation of *C. burnetii*. Other cell lines, e.g., epithelial lining (Vero E6), macrophages (P388D1, J774, DH82) and murine fibroblastic cells (L929), are also used [9,42,43]. After inoculation, centrifugation is performed for 1 h at 700× *g* so that the bacteria properly stick to the cells. Three shell vials are used for the same inoculum. At day 3, 10 and 21 post-inoculation, *C. burnetii* vacuoles can be seen under an inverted microscope. After 10 days, proliferating *C. burnetii* inside the cells are detected directly on the coverslip within the eggshell vial by direct immunofluorescence assay (IFA) using polyclonal anti-*C. burnetii* antibodies and a secondary antibody conjugated to fluorescein isothiocyanate (FITC). Cells present in the remaining shell vial are harvested and incubated at 37 °C for 2 months in 5% CO_2_, which may be extended up to 4–5 months. During incubation, a change of culture medium once a week and periodical evaluation of bacterial growth using either light or fluorescence microscopy is required. In the case of cytopathogenic effects (CPE) or PCR giving positive results, subcultures are carried out. Supernatant is inoculated on confluent layers of Vero cells or fibroblasts (L929) in a 150 cm^2^ culture flask to obtain new bacterial isolates. Although this method was established for human samples, it can be used efficiently for animal samples [9,20,31].

#### 1.2.3. Laboratory Animals

Animal inoculation is a useful technique for isolation and propagation of *Coxiella* obtained from contaminated samples such as ticks or those obtained from animals, e.g., feces, milk, vaginal discharges and fetal parts of the placenta. Laboratory animals act as a “filtration system” for such samples. Mice and guinea pigs are commonly used for this purpose. Following intraperitoneal inoculation with a dose of 0.5 mL of suspension (1:10) per animal, body temperature and antibody titer can be examined. This protocol should be carried out in conjunction with serological assays on other laboratory animals (mice and guinea pigs) that have been inoculated with the same samples. Sera are collected 21 days post-inoculation. A positive result confirms the diagnosis of *C. burnetii* infection. The results can be confirmed further through (real-time) PCR or by microscopy, using impressions and stained samples of collected spleen, liver and lungs. Splenomegaly is a typical sign caused by substantial growth of *C. burnetii*. Spleen, liver or lung samples are then further inoculated into embryonated chicken eggs or cell culture systems for isolation of *C. burnetii* [20,31,43,44].

#### 1.2.4. Axenic Media

Inoculation of axenic medium complex *Coxiella* medium (CCM) is a novel technique used for isolation and growth of *C. burnetii* [45]. Recently used axenic medium is an acidified citrate cysteine (ACC) medium. The actual formulation, designated as defined acidified citrate cysteine medium (ACCM-D), allows replication of *Coxiella* over 14 days with morphological differentiation (SCV/LCV). In this medium, antimicrobial factors such as proteolytic and hydrolytic enzymes and an acidic environment similar to that present in the acidic phagolysosome of antigen presenting cells are provided. Hence, axenic medium provides an ideal environment for the growth of *C. burnetii*. This medium is quite useful for genotypic and phenotypic characterization of *C. burnetii* variants [20,45,46]. According to a propidium monoazide (PMA)–PCR based study, there is no difference in viable cell count obtained through the cell-free system (axenic medium) and the cell-based culturing system. Moreover, axenic medium does not influence the livability of cells, relative virulence and antigenic phase variation of *C. burnetii* as compared to cell-based culturing systems [30].

### 1.3. Genome and Genetic Characterization

Genetic and molecular characterization of *C. burnetii* is useful for surveillance purposes and for epidemiological investigations of the outbreaks. It is also a useful to investigate the genotypic variation of a pathogen concerning geographical area and to explore interactions between various types and subtypes of the bacterium [47,48,49]. This information helps in control program planning for potential reservoirs involved in the life cycle of *C. burnetii* [18,47,50]. 

Several techniques are used for molecular typing of *C. burnetii*, including pulsed-field gel electrophoresis (PFGE), sequence analysis or restriction fragment length polymorphism (RFLP) of single genes. All of these techniques have limitations such as poor discriminatory power and an inappropriate reproducibility and transferability [48]. However, two recent PCR-based typing techniques, i.e., multilocus variable number of tandem repeats analysis (MLVA) and multispacer sequence typing (MSST) possess high discriminatory power and are easily reproducible. MSST is based on variation in 10 short intergenic regions and can be conducted directly on DNA extracted from clinical or environmental samples without isolation of the bacteria. Both techniques allow identification/differentiation of up to 36 genotypes of *C. burnetii* [36,51,52].

The first whole genome sequencing of *C. burnetii* from the Nine Mile RSA 493 reference strain, obtained from an infected group of *Dermacentor andersoni* in 1935, was released in 2003. Random shotgun technique was used for this sequencing, which was a 1,995,275 base pair lane. In 2007, another genome sequencing report was published using the Henzerling RSA 331 strain obtained from blood of an infected patient in Italy in 1945. The genome of *C. burnetii* is circular in shape, with about 1.9 to 2 Mbp. One of the following five plasmids: *QpDG* (51 kb), *QpRS* (39 kb), *QpH1* (36 kb), *QpDV* (33.5 kb) and the plasmid of Chinese isolate (56 kb) or a chromosomal integrated plasmid associated sequences (16 kb) can be found. The presence of a high number of pseudogenes in the genome of *C. burnetii* indicates that the bacterium undergoes genome reduction [20,39,53]. The whole genome sequencing (WGS) technique is becoming more affordable. However, data interpretation remains time consuming, and it requires special expertise, technical skills, bioinformatics knowledge and additional funding. The most communal MLVA genotype A found in animal species is found in caprine, ovine, rats and environmental samples. The presence of this highly prevalent genotype A in humans, environment and animals confounds the discovery of an accurate source of infection [20,54]. 

### 1.4. Transmission

The major route for acquiring *C. burnetii* infection is by uptake of a contaminated aerosol, while consumption of contaminated raw food materials, e.g., milk, etc. is the minor source of transmission. Occasionally, the infection may occur after skin or mucosal contact with contaminated products, blood transfusion or mating [14,29,55]. However, in animals, ticks may play an active role in disease transmission [56]. Body secretions and excretions, e.g., milk, saliva, parturition products, aborted materials, urine and feces, contain a large number of *C. burnetii*, which may result in sexual and vertical transmission of the disease. These discharges can dry and combine with dust, ultimately leading to human exposure through aerosols [37,57]. Aerogenic transmission of the disease from contaminated sites to humans depends on atmospheric dispersion and the impact of environmental factors on deposition and re-aerosolization [52]. Although human-to-human transmission of Q fever is rare, it may occur following contact with parturient women. Transplacental transmission, cutaneous inoculation and postpartum spread of *C. burnetii* do occur in sporadic cases. The disease is reported in more than 40 species of ticks from the families Ixodidae and Argasidae, and some other arthropods that feed on animals [37,57]. Transmission of *C. burnetii* infection through a tick bite is still dubious in humans. However, ticks can transmit *Coxiella* both transovarial and transstadial to their offspring, thus acting as potential reservoir. Infected ticks excrete large amounts of *Coxiella* in their feces, which contaminate the skin of host animals. Thus, ticks are important for environmental spread of *Coxiella* infection [27,58,59,60].

### 1.5. Occurrence of Coxiella burnetii in Different Body Fluids and Tissues

Shedding of *C. burnetii* can occur in different body tissues and fluids such as milk, feces, urine, birth fluids, vaginal secretion and fetal membranes. Particularly, in the case of reproductive failure, a large quantity of bacteria is shed through vaginal secretions and birth fluids [10,61]. It is reported that approximately 1 billion *C. burnetii* per gram of placenta are excreted in birth fluids of an aborted animal [8]. Similarly, placenta of seropositive sheep and goats without symptoms can contain more than 10^9^ hamster-infective doses of *C. burnetii* per gram of tissue, although a single bacterium is enough to cause Q fever infection [7,8,62,63]. 

The organism is shed in body fluids for a variable period, depending upon host species and shedding routes. Infected cattle can persistently shed pathogens in their milk for several months without any clinical signs or symptoms, while shedding through vaginal mucus or feces is sporadic or intermittent in nature [64]. Real-time qPCR is useful to determine the load of bacteria in vaginal and milk samples. During the acute phase of Q fever, 10^4^–10^8^ *C. burnetii* bacteria were found in vaginal swabs, while 10^2^–10^6^ bacteria were present per milk sample. Shedding of pathogens declined continuously within two months to less than 10^4^ bacteria per vaginal swab and 10^2^ per milk sample. At the end of this study, a 10-fold increase in bacterial shedding was reported [65,66]. Seropositive animals may not secrete the organism. Similarly, some apparently healthy animals may shed the organism even if they are seronegative [20,67].

Prevalence of *C. burnetii* in milk samples obtained from various ruminant animals such as sheep, goats and cattle may vary due to differences in shedding routes. The principal routes for bacterial shedding in sheep are feces and vaginal fluids, while these are minor routes in cattle. Milk and blood are not the common routes for bacterial shedding in sheep. In cattle, milk is considered the major route for bacterial shedding. Goats can excrete organism through vaginal mucus, feces and milk. However, major routes for bacterial shedding in caprine species are feces and blood, not the milk. Feces contain the highest numbers of bacteria in goats [7,68]. 

### 1.6. Pathogenesis

*C. burnetii* possesses a distinct characteristic called phase variation of the cell wall. Phase I bacteria have a complete LPS molecule and are highly virulent. This virulent form of bacteria can be isolated from infected animals, human beings and ticks. However, Phase II bacteria are avirulent and can be obtained after serial passages of Phase I bacteria in cell culture or embryonated chicken eggs. LPS of Phase II is rough and truncated. Besides LPS, the two antigenic forms of *C. burnetii* also differ in cell density, surface charge and surface protein configuration [39,69]. Morphologically, there are two different forms of *C. burnetii*: the large cell variant (LCV) and the small cell variant (SCV). The LCV is larger in size with a less electron-dense center, while SCV is a metabolically inactive and less replicating form, with a compact rod shape and dense central region. These SCVs are excreted by infected animals, leading to environmental contamination [36]. 

The main route for *C. burnetii* transmission in both animals and humans is inhalation and to a lesser extent by ingestion of contaminated milk and milk products [19]. Once the organism enters the body, it attaches to the cell membranes of phagocytes (monocytes/macrophages). Attachment of virulent bacteria to the phagocytic cells is triggered by avb3 integrin, while for avirulent bacteria, avb3 and complement receptor CR3 mediate the attachment. Phase I bacteria survive inside the phagocytic cells, whereas Phase II bacteria are eliminated. Additionally, Phase I bacteria are phagocytosed by the host cells in a considerably lower amount than Phase II bacteria [70]. 

The SCVs are phagocytosed by monocytes and macrophages, and enter the phagolysosomes. Here, SCVs fuse with the lysosomal contents, change into the metabolically active form, undergo vegetative growth and ultimately transform into LCVs. Normally, both antigenic forms of *C. burnetii* are present within this phagolysosome niche. However, Phase II bacteria are quickly eliminated. The acidic environment of phagolysosomes is highly conducive for the growth of *C. burnetii*. Most important is the organism’s ability to propagate and multiply within the acidic phagolysosome and its tendency to develop persistent infection. The entire developmental cycle of a metabolically active Phase I bacterium occurs within this acidic niche [19,36,71,72]. This acidic pH ensures the availability of nutrients essential for growth of *C. burnetii* and protects it from the effect of various antimicrobials [70]. 

Little is known about the role of the host cellular immunity in infected human patients. The goat’s immune response against *C. burnetii* infection revealed that both IgG and IgM Phase II specific antibodies can be found within two weeks post-infection and their titers remain elevated in the blood for up to 13 weeks. Phase I antibodies develop after four weeks of Phase II antibodies. The duration of immune response against *C. burnetii* can persist for several months to years. The metabolically active LCVs are mainly present in the trophoblasts of the placenta [73]. During acute infection, the organism is present in blood, liver, spleen and lungs of the host. The disease is mostly asymptomatic in nonpregnant animals, while in pregnant animals the most important clinical manifestations are abortion, stillbirth, birth of weak offspring and premature delivery. Incidence of respiratory and digestive problems in apparently healthy kids in at-risk areas can be associated with Q fever infection. Although reproductive disorders are not the common consequences of Q fever in domestic animals, increased abortion rates of up to 90% have been reported in goats [14,73,74].

In human beings, *C. burnetii* infection appears both as an acute and chronic infection. Acute infection is often self-limiting with mild flu-like symptoms, while chronic Q fever is life threatening with chronic endocarditis in many cases [75]. In abortions due to *C. burnetii* infection, fetuses usually look fresh and normal; however, sometimes fetuses are necrotic. Macroscopically, there is inflammation of placenta with purulent yellow-brownish exudate in severely affected intercotyledonary spaces. Microscopically, the trophoblastic cells present at the base of villi and in the intercotyledonary area of the allanto-chorion are mostly affected. This inflammation may vary from mild mononuclear infiltration to chronic necrosis with pus-like discharge. The epithelial cells present in chorionic membranes at the base of villi often have basophilic intracytoplasmic granulation and a foamy vacuolated cytoplasm. Histopathological examination of some fetuses showed inflammation of liver with mild granulation. However, other organs were found apparently normal [73,76].

### 1.7. Clinical Signs and Symptoms 

#### 1.7.1. Humans

In humans, the clinical nature of this disease is highly variable. It can lead to an acute infection characterized by mild febrile illness, pneumonia and hepatitis, while in rare cases chronic disease may develop in the form of endocarditis, and abortion and stillbirth in pregnant women. The fever due to *C. burnetii* infection is remittent and usually persists for 9–14 days. It is considered as a self-limiting disease [51,75]. Approximately 60% of the infected people remain asymptomatic while about 40% of the patients show clinical signs. As far as chronic Q fever is concerned, it develops in 3–5% of patients mostly in the form of endocarditis. Patients with valvular disorders, microbial arteritis, vascular implants and immunocompromised persons are more prone to this infection [77]. The most common manifestations of Q fever are mild flu-like symptoms with sudden increase in body temperature, restlessness, excessive sweating, severe headache, myalgia, arthritis, anorexia, upper respiratory tract problems, persistent cough, pleuritic chest pain, chills, confusion and GIT problems such as nausea and diarrhea. Another important symptom of this disease is Q fever fatigue syndrome (QFS), which is a debilitating condition following acute Q fever involving main body systems. It occurs in approximately 20% of patients. Although this persistent fatigue is not a life-threatening condition, it results in serious social and economic consequences in the form of loss of person’s quality of life and inability to work. QFS was thought to be the major cause of Q fever associated economical losses during the Dutch Q fever outbreak (2007–2010). In rare cases, an acute Q fever develops into chronic infection. Endocarditis is the main clinical manifestation of the chronic form. Other complications such as pericarditis, myocarditis, thyroiditis, osteomyelitis, nephritis, meningoencephalitis, hemolytic anemia, hemophagocytic syndrome, severe cephalgia and retro-orbital pain are rare manifestations of chronic infection [74,77,78,79]. During pregnancy, infection is usually asymptomatic. However, some serious obstetrical disorders such as placentitis, spontaneous abortion, fetal growth retardation, stillbirth, premature delivery and birth of weak offspring have been reported. Infection during pregnancy may also result in abortions during subsequent pregnancies. However, a recent study conducted in an area with the highest number of Q fever outbreaks in the Netherlands found no association between Q fever and adverse pregnancy outcomes [7,80]. *C. burnetii* infection results in high morbidity and low mortality. Mortality has been reported in 1–11% of the chronic Q fever patients [8]. This infection causes serious long-term complications on patient’s health and social life due to long-lasting Q fever fatigue syndrome [81]. The economic losses caused by the Netherlands Q fever outbreak (2007–2010) were estimated to be approximately 0.307 billion EUR [79]. 

#### 1.7.2. Animal Infection

In animals, Q fever is often called coxiellosis and usually occurs without any apparent clinical signs. It is not considered a veterinary health problem except in (small) ruminants, where *C. burnetii* is a well-known cause of abortion. Ruminants, especially sheep and goats, are major reservoirs of *C. burnetii*. The historical Q fever outbreak in the Netherlands was linked with infected small goat farms in close vicinity to residential areas [61,82]. In animals, especially small ruminants, Q fever usually results in reproductive problems such as spontaneous abortion during late pregnancy, premature delivery, stillbirth and birth of weak offspring, along with infertility in cattle [8,62]. In cattle, acute Q fever usually appears as subclinical infection, while chronic infection may result in reproductive disorders. There is no reliable evidence which proves that Q fever causes retention of fetal membranes, subfertility, metritis or endometritis in cows [62]. Abortion due to *C. burnetii* infection usually ranges from 3–8% [79]. During the recent Dutch Q fever outbreak, up to a 60% abortion rate was recorded in pregnant goats during final month of pregnancy, with no apparent signs of illness. Endometritis was reported in some goats with previous history of abortion. Full-term kids were emaciated with lower body weight and high mortality. Several other apparently healthy kids showed respiratory and digestive tract problems [83]. In dairy animals, *C. burnetii* infection may lead to subclinical mastitis. The organism resides in mammary glands and placenta of pregnant dairy animals with fetus and associated structures having the highest amount of *C. burnetii*. Thus, post-parturient shedding of organisms in birth fluids is higher in small ruminants, while lower in the case of cattle [32,62]. 

### 1.8. Diagnosis

Diagnosis of Q fever based on clinical signs and symptoms or post-mortem examination is almost impossible because of nonspecific clinical presentation or missing symptoms and lesions of the disease [84]. Thus, for accurate diagnosis of *C. burnetii* infection, laboratory evidence is obligatory. Generally, four categories of diagnostic techniques are available for Q fever diagnosis: (i) isolation and propagation of the organism, which requires BSL-3 laboratory using tissue culture, embryonated chicken eggs or laboratory animals; (ii) serodiagnostic tests including IFA, CFT and enzyme immunoassay; (iii) antigen detection assay such as immunohistochemical staining (IHC); and (iv) genomic detection assays such as PCR. Use of various laboratory tests in combination, i.e., ELISA for serology and PCR for nucleic acid detection, is highly suggestive for confirmatory Q fever diagnosis [37,84]. In ruminants, both IFA and ELISA are suitable techniques for serological investigation of Q fever, while in humans IFA is considered as an ideal technique for Q fever diagnosis because of its high sensitivity and specificity [85,86].

#### 1.8.1. Serological Tests

Serodiagnostic techniques are preferable for Q fever diagnosis as isolation techniques remain expensive, laborious and less sensitive. Serology is best utilized to estimate herd-level prevalence of the infection in animals. In humans, serology can recognize the difference in antibody titers in acute and chronic infection, which makes it a choice for diagnosis of Q fever [64,67]. In acute infection, IgG antibody titers are higher against Phase II antigen only, while in chronic form, both IgG and IgA antibody titers are high against both Phase I and Phase II of the bacterium. However, overlapping of antibody titers in both acute and chronic forms is possible [52,69]. However, serological results remain questionable in carrier animals, window infection period and in endemic and post-epidemic infection status [84,87].

A. Immunofluorescence Assay (IFA)

Immunofluorescence assay is a reference method for detection of anti-*C. burnetii* antibodies in human sera. It is very useful, especially for follow-up of the patient’s disease status and to identify patients with risk of developing chronic infection. This assay can easily differentiate between suspected acute and chronic forms of Q fever by measuring Phase I and Phase II antibody titers. If the Phase I antibody titer is ≥ the Phase II titer, it will indicate a chronic form of Q fever, and if Phase II antibody titer is > the Phase I titers, the result is indicative for an acute infection. IgG antibody titers of ≥1:800 against Phase I antigen is indicative for Q fever endocarditis. This technique is commonly used for diagnosis of Q fever in humans, while in animals no commercial Q fever IFA kit is yet available [36,88,89]. Although IFA is considered as the gold standard for diagnosis of Q fever in humans, it has limitations. This technique is not suitable for detection of early acute Q fever because of the lag in antibody titer development (7–15 days after the onset of clinical disease). It requires very specific and expensive instruments along with a high level of expertise for proper interpretation of results. The species-specific IFA cannot be used for extensive or herd-level screening. That is why IFA, which is the gold standard technique for Q fever diagnosis in humans, is not used for routine detection of *C. burnetii* infection in animals [36,90].

B. Complement Fixation Test (CFT)

The World Organization for Animal Health (OIE) reference serological assay list regards the complement fixation test as a reference assay for Q fever diagnosis but, currently, its use is infrequent because of its lower sensitivity. Another drawback of CFT is the presence of anticomplementary activity in several samples, which hinders antibody titer estimation even after repeated attempts. Similarly, anti-*C. burnetii* antibodies present in serum samples of sheep and goats cannot be regularly detected by IFA antigen [32,36].

C. Enzyme-Linked Immunosorbent Assay (ELISA)

Enzyme-linked immunosorbent assay method is a more specific and sensitive technique than any other serological assay and is indorsed by the European Food Safety Authority (EFSA) for test harmonization. Particularly, in the case of animals, it is preferred to IFA and CFT because of its convenience for herd- or flock-level screening and its ability to detect *C. burnetii* antibodies in various animal species. IDEXX reported 100% sensitivity and specificity of their ELISA kit [14,36,39]. An ELISA using antigen from ruminant isolates is more sensitive than an assay with antigen from ticks. Thus, EFSA recommends ELISAs containing *C. burnetii* antigen from ruminants’ isolates. ELISA can detect antibodies in serum against both antigenic phases of *C. burnetii* and provides a cumulative interpretation of results as seropositive, suspect or seronegative status [36,91]. 

#### 1.8.2. Staining

In this technique, stained tissue or vaginal mucus smears are observed under a microscope with an oil immersion objective lens for detection of the causative agent. *C. burnetii* is an acid-resistant bacterium. Different kinds of stains such as Stamp, Gimenez, Machiavelli, Giemsa, modified Ziehl–Neelsen and modified Koster staining can be used [31]. The first three stains give the best results. However, due to lack of specificity, a positive result is only a presumptive indication of *C. burnetii* infection. Therefore, further investigations should be carried out for confirmatory diagnosis. 

#### 1.8.3. Polymerase Chain Reaction (PCR)

Polymerase chain reaction is used for molecular detection of *C. burnetii*. It is a rapid, highly specific and sensitive diagnostic tool compared to all other laboratory techniques. The higher sensitivity of PCR to detect and quantify small concentrations of bacterial DNA has significantly improved diagnostic and research approaches [36,92]. PCR can be performed on a variety of biological specimens, such as fetal membranes, fetal fluids, genital swabs or samples from aborted fetuses (liver, lung or abomasal contents). Blood, serum, milk, urine, anal and throat swab specimens are also useful for genomic detection of *C. burnetii* using qPCR [31,36,92]. In the case of chronic infection, samples can be obtained from focal tissue samples of infected organs such as valvular material for endocarditis or aneurysm, vessel fragments in the case of vasculitis and bone biopsy in arthritis. As the antigen is shed intermittently in urine, feces, vaginal discharge and milk, it is preferable to investigate consecutive samples for genomic detection of the pathogen [20,84]. PCR targeting the insertion sequence *IS1111*, a repetitive transposon-like element of *C. burnetii*, is highly sensitive and specific for genomic detection. However, *IS1111* cannot be used for quantification of *C. burnetii* DNA because of having multicopies up to (20 copies per genome), and misidentification with *Coxiella*-like organisms. Single copy genes such as *icd* and *com1* are useful for quantification of *C. burnetii* DNA. Different pairs of primers targeting various target genes such as superoxide dismutase (*sodB*), isocitrate dehydrogenase (*icd*), *com1*, macrophage infectivity potentiator protein (*cbmip*), heat shock proteins including *htpA* and *htpB*, and some plasmid mediated genes such as *QpRs*, *QpH1* and *cbbE* can be used for the detection of *C. burnetii* DNA [36,93]. The best time for PCR assays to detect *C. burnetii* DNA in blood or serum sample are first two weeks after onset of clinical infection. During this period, there is a lag in antibody titer development, and serological tests are useless during this period. However, the PCR test can be used successfully to detect *C. burnetii* DNA in blood or serum samples during this interval. Two weeks after the onset of clinical signs, IgG antibodies titer starts developing and at the same time *C. burnetii* DNA becomes undetectable in the blood. Hence, serological techniques can be best utilized two weeks after onset of the clinical infection [84,87]. The quantitative real-time PCR can successfully be utilized to detect *C. burnetii* shedders. Once the seropositive animals are detected in a flock with serological assays, the PCR is then a technique of choice to trace the shedders [84]. 

### 1.9. Prevention and Control

Disease surveillance, regular monitoring and implementation of proper preventive and control strategies are necessary to reduce further disease outbreaks in an area. These strategies pose economic and public health significance in reducing reproductive losses in the livestock industry and to avoid potential risk of transmission of infection to human beings. Determination of herd-level prevalence of a disease in a population could help in proper planning and implementation of preventive and control measures [81,83,94]. Because of the self-limiting nature of this disease, the prevalence of Q fever usually declines with time without adopting any control strategies. This may be because of natural immunization of hosts against *C. burnetii* [36]. Preventive vaccination, manure management including covering and compositing of manure or treating manure with lime, better livestock farm and wool-shearing practices, use of isolated calving pens, restrictions on free animal movement, and proper disposal and burial of aborted materials are important measures to prevent the spread of *C. burnetii* infection. Hygienic practices, especially calving pen cleanliness, is considered an important measure in preventing this infection. Similarly, disinfection of calving pens, naval cord disinfection, and proper disposal of aborted fetuses and fetal membranes, and provision of new bedding at the time of calving are important measures to reduce the risk of disease transmission. Birth products including fetal membranes and dead fetuses should immediately be disposed to avoid their ingestion by stray dogs, wild carnivores and even domesticated animals, which may also spread the infection in the environment [32,73,94]. Furthermore, quarantine measures should be implemented at livestock farms, and animals from infected flocks should not be mixed with healthy animals at the farm. Raw milk from infected dairies should not be used for drinking or any other purpose, because large numbers of bacteria are shed in the milk of infected animals. Seropositive animals shedding the organism into the environment are an important source of disease transmission. It is important to identify such shedders and remove them from the flocks [8,64]. 

Training and awareness of livestock-associated professionals and farmers are important in reducing the risk of disease spread. Individuals working on disease surveillance should adopt personal protective measures such as protective clothing (including FFP-3 breathing masks), protective gloves and disinfection of sampling materials immediately after use. Similarly, all consumables should be properly discarded after use [83]. The distance between residential areas and livestock farms, especially those containing pregnant ewes, should not be less than 500 m to reduce the risk of disease transmission. To prevent farm-to-farm spread of infection, animal producers should avoid transporting and marketing animals, especially periparturient animals, during ongoing abortion outbreaks [21,95]. As Q fever is a cosmopolitan zoonosis, interdisciplinary cooperation among medical doctors, veterinarians, laboratory working groups and farmers is required to understand how this pathogen circulates in a geographical area and to plan strategies for its proper control and prevention [96].

Vaccination is followed by an active immunological response against the potential pathogen. The use of anthelminthic drugs prior to vaccination is useful to gain an improved immunological response [97]. Currently available inactivated Phase I vaccine for animals containing Nine Mile RSA 493 strain of *C. burnetii*, which was isolated from ticks, is recommended by OIE in Q fever endemic areas. This vaccine is reported to cause reduction in abortion rates, decrease in bacterial shedding and reduces the risk of disease transmission to humans. However, it is less effective in outbreak situations compared to regular vaccination [36]. Use of an inactivated Phase I vaccine (Coxevac^®^, Ceva Santé Animale, Libourne, France) in noninfected sheep and goats prior to their first breeding results in reduced abortion rate and bacterial shedding. Some studies reported that use of Coxevac during pregnancy also reduces bacterial shedding, although this vaccine is not approved for use in pregnant animals. Inactivated Phase I vaccine was proven to be very effective in Dutch small ruminant flocks after accomplishment of an extensive vaccination campaign in 2010. Since then, no *C. burnetii* borne abortion has been reported from vaccinated flocks, and a gradual decline occurred in the number of PCR positive farms based on bulk tank milk (BTM) sampling [73]. Performing repeated annual vaccination in susceptible herds is recommended, especially for young animals, in at-risk areas [31]. Phase I vaccine is equally effective in humans as in the case of animals, but it is contraindicated in individuals already exposed to *C. burnetii* infection, e.g., Q-VAX^®^ (Seqirus, Parkville, VIC, Australia) is the only available Q fever vaccine in Australia for human use. Q-VAX^®^ is an inactivated Phase I whole-cell vaccine containing the Henzerling RSA 331 strain of *C. burnetii* isolated from blood of a Q fever patient in Italy. In Australia, Q-VAX^®^ vaccination is recommended on routine basis in individuals occupationally exposed to Q fever infection [31,98]. 

### 1.10. Treatment

#### 1.10.1. Treatment in Humans

There are two forms of Q fever in humans, i.e., acute and chronic. Antibiotics are effective against the acute form of this disease, but once the infection proceeds to its chronic form, then treatment time is prolonged and recurrence of the disease is usual, which may lead to high mortality. Duration of antibiotic therapy is established based upon follow-up of serological titers in a Q fever patient [90,99]. The antibiotic treatment must be started immediately after the onset of clinical disease because delayed antibiotic treatment may not be effective [100]. Generally, acute Q fever is self-limiting. However, timely detection and antibiotic administration may decrease the duration of infection and severity of symptoms. The drugs of choice for Q fever are doxycycline and hydroxylchloroquine. These drugs are mostly used in combination. Other antibiotics, such as erythromycin, rifampin, roxithromycin and clarithromycin, can be used as alternative therapy [8,99,101]. A dose of 100 mg doxycycline two times a day for 2 to 3 weeks is recommended for acute Q fever patients, especially nonpregnant women and adult patients. Hydroxychloroquine can also be used in combination with doxycycline. Hydroxychloroquine is a lysosomotrophic drug that increases phagolysosome pH. As hydroxychloroquine elevates the phagolysosome pH, it acts as bacteriostatic because *C. burnetii* requires an acidic environment for its multiplication [8]. In the case of pregnant women and children <8 years of age, cotrimoxazole can be used safely for treatment of Q fever [69]. 

In the case of chronic Q fever, especially native and prosthetic valve endocarditis, antibiotics such as doxycycline and hydroxychloroquine can be used effectively at a dose rate of 200 mg per day but for a longer period of 18 to 24 months. Combination therapy consisting of these two antibiotics is more effective in preventing the development of endocarditis than doxycycline alone. Rifampicin, macrolides and quinolones are less effective against *C. burnetii* infection; therefore, they are not usually used as alternative treatment for this disease [55,69]. Methotrexate is an important steroid replacement used to suppress vascular inflammation and to maintain homeostasis of ascending and thoracic aorta [55]. Follow-up care such as regular heartbeat and eye-reflex examinations are necessary after antibiotic treatment. Photosensitivity may be a problem in some patients after antibiotic use. In advanced stages of chronic Q fever, characterized by severe cardiac failure or abscess formation in heart valves, use of antimicrobials is not favorable. In these situations, cardiac surgery is recommended [69,88]. The use of interferon (IFN) and tumor necrosis factor (TNF) for chronic Q fever treatment has also been proven effective [8,69]. In the case of chronic infection, the follow-up of serological response is necessary, and the treatment can be stopped when phase I IgG antibodies titer declines by at least four-fold. Special attention should be given to individuals more prone to Q fever infection because the infection can lead to high morbidity and mortality if left untreated [99,102]. 

#### 1.10.2. Treatment in Animals

Limited information is available about the treatment of coxiellosis in animals. Extensive data are required to determine the efficacy of antibiotics in preventing bacterial shedding and reproductive losses in animals due to *C. burnetii* infection. Usually, tetracycline is recommended for treatment in animals, but usage of tetracycline in animal feed during gestation period, as a herd-level disease control strategy, is not effective because of its reduced bioavailability following oral administration. Parenteral use of two injections of (long-acting) oxytetracycline 20 days apart at 20 mg/kg during ongoing coxiellosis abortions may be useful in preventing reproductive losses to the animals. However, oral administration of oxytetracycline is not effective in reducing bacterial shedding with birth fluids and in altering the serological status of animals [103]. In ruminants, tetracycline administration with an interval of 2–3 weeks in pregnant animals from 95th day of gestation until parturition is effective in reducing the risk of abortion due to other pathogens such as *Chlamydophila abortus* [83].

### 1.11. Neglected Zoonosis in Pakistan

In Pakistan, data on coxiellosis and Q fever remain neglected. To our knowledge, from 1955- to date, only twenty-four reports were found describing the prevalence of *C. burnetii* infection in humans and animals for this country. According to these studies, the prevalence of coxiellosis ranged from 4.6% to 40% in all livestock species and 10.2% to 26.8% in humans [56,92,104,105,106,107,108,109,110,111]. Q fever is not a notifiable disease in Pakistan [112]. Recent reports in small ruminants, camels and humans highlight the need of attention toward this disease, especially in those areas where animal–human interaction remains common and milk transportation chains are not up to date [92,104,105,109,113]. *Coxiella* DNA was confirmed in soils of Punjab, which further points out the importance of the “One Health” concept in combating this disease [105]. Calls have been made to prioritize such zoonotic diseases in Pakistan under the “One Health” paradigm [114]. Awareness programs will help in this scenario, both within the general population and the scientific communities of Pakistan and neighboring countries [92,105,115].

### 1.12. Conclusions

Q fever remains a neglected zoonosis in many developing countries of the world including Pakistan. *C. burnetii* can sustain longer to harsh environmental conditions. Moreover, asymptomatic infections in animals could threat human health, thus, posing a serious “One Health” risk. Pathobiological studies including host-pathogen relationships, epidemiology and genomic information would help understanding and identifying the potential, outbreaks and evolutionary-relationships of the pathogen from different sources. Ensuring pasteurization of the milk before consumption and following protective measures while handling animals can help infection preventing in humans. Awareness programs would be helpful to create acceptance of these measures in human populations.

## Data Availability

Not applicable.
